# Supplementary material for: Spontaneous and Ionizing Radiation-Induced Aggregation of Human Serum Albumin: Dityrosine as a Fluorescent Probe
Source: Int J Mol Sci. 2022 Jul 22;23(15):8090. doi: 10.3390/ijms23158090 (PMC9331647; doi:10.3390/ijms23158090)
Supplement: Supplementary file 1 [file ijms-23-08090-s001.zip › ijms-1832423-supplementary.pdf]

Supplementary Materials

Karolina Radomska, Marian Wolszczak *

* Correspondence: marian.wolszczak@p.lodz.pl

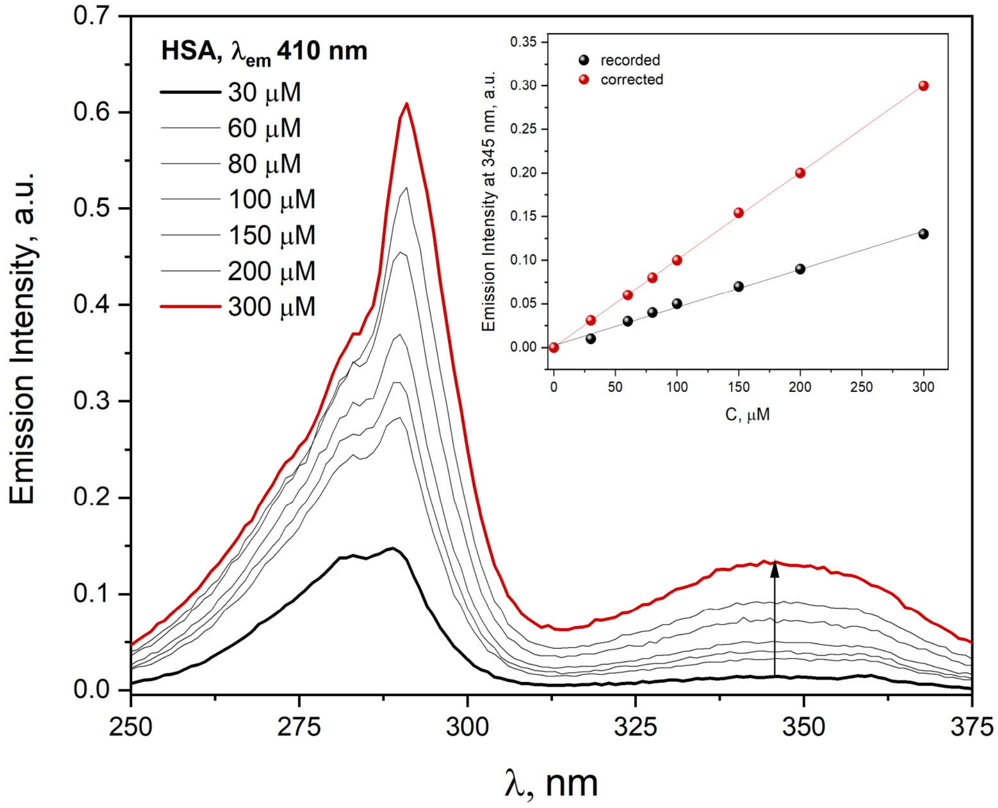

Figure S1. Emission excitation spectra of the neat HSA solution (30-300 μM). The emission wavelengths were 410. **Insert**: Dependence of I_{max} as a function of human serum albumin concentration (30–300 μM). I_{max}–intensity of HSA emission in buffer solution at maximum of excitation emission band, λ_{em}=410 nm. The red dots represent the I_{max} after correction for inner filter effect.

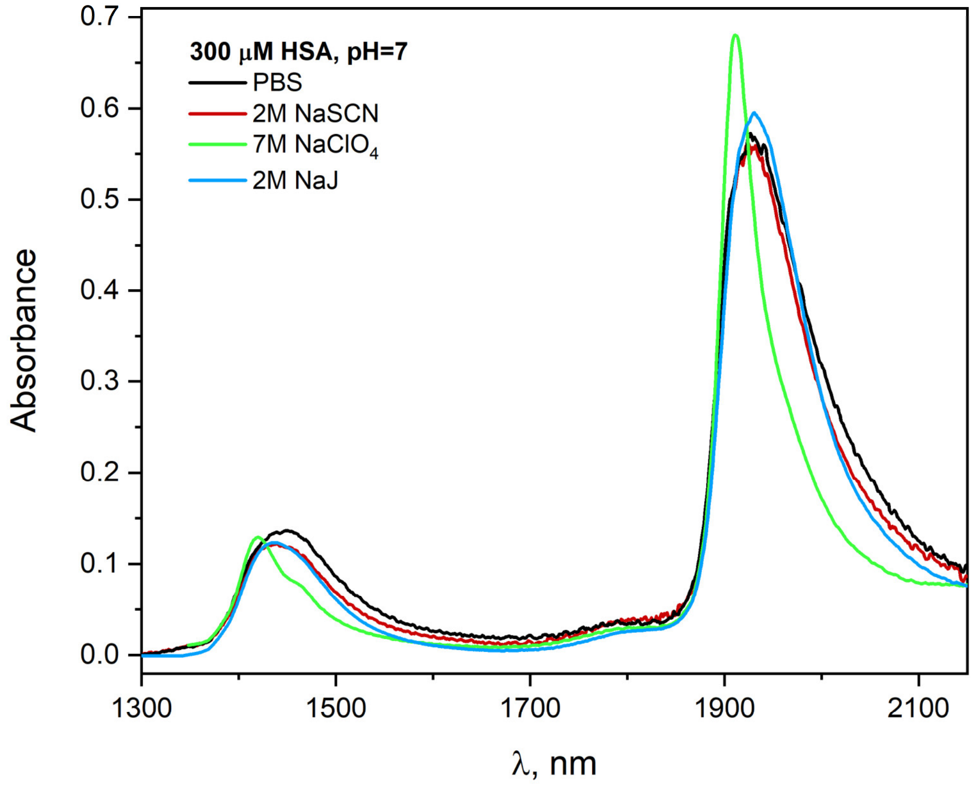

Figure S2. Absorption spectra of buffer solution of HSA (300 μM) containing NaSCN (2M), $NaClO_4$ (7M), NaJ (2M). Measurements were made using a 0.1 mm quartz cuvette. The PBS plot represents the spectra of HSA (300 μM).

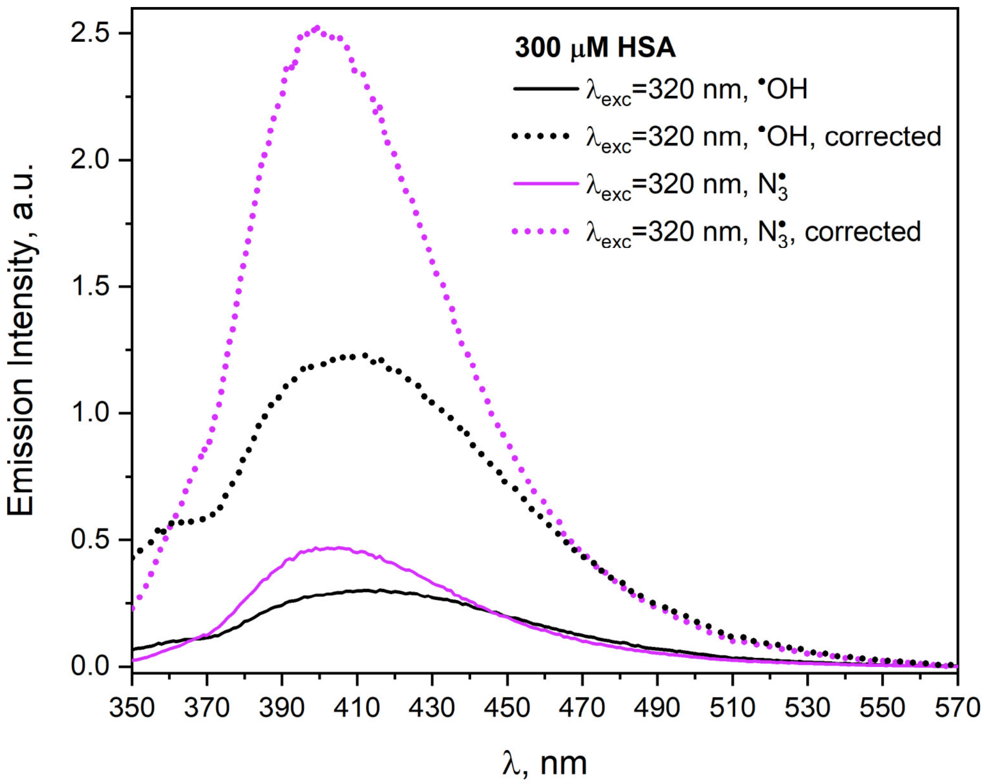

Figure S3. Emission spectrum of N_2O-saturated buffer HSA solution (300 μM) containing NaN_3 (0.1M), obtained for irradiation dose 27.8 kGy (violet curve). Emission spectrum of N_2O-saturated buffer solution of HSA (30 μM), obtained for irradiation dose 27.8 kGy (black curve). The excitation wavelength was 320 nm. The dotted curves represent the HSA emission spectra after correction for inner filter effect.

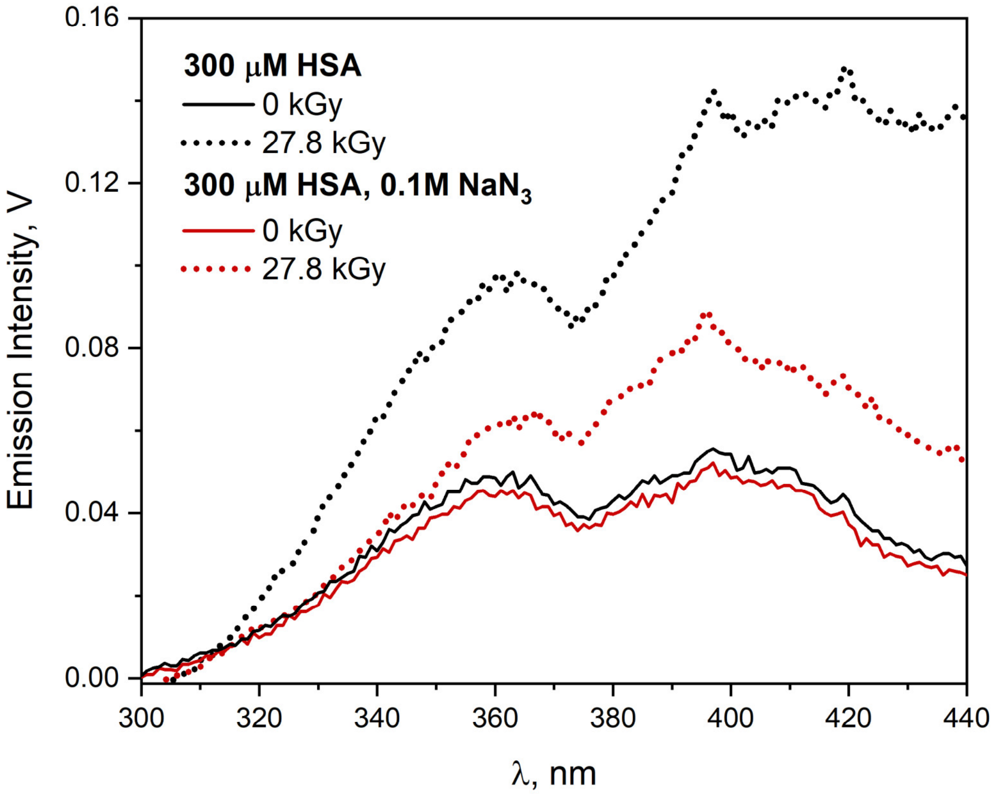

Figure S4. Emission excitation spectra of the N_2O-saturated HSA solutions (300 μM): neat or containing 0.1M NaN_3, obtained for irradiation dose 27.8 kGy. The emission wavelengths was 525 nm.

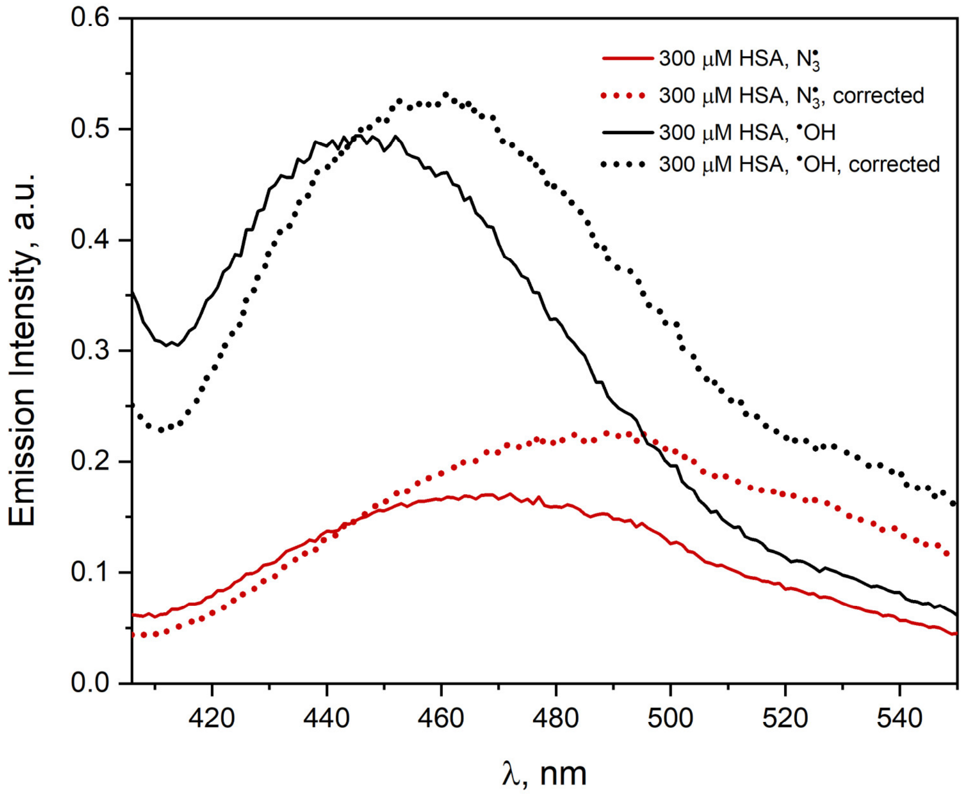

Figure S5. Emission spectrum of N_2O-saturated buffer HSA solution (300 μM) containing NaN_3 (0.1M), obtained for irradiation dose 27.8 kGy (red curve). Emission spectrum of N_2O-saturated buffer solution of HSA (30 μM), obtained for irradiation dose 27.8 kGy (black curve). The excitation wavelength was 390 nm. The dotted curves represent the HSA emission spectra after correction for instrument response (mainly photomultiplier spectral response).

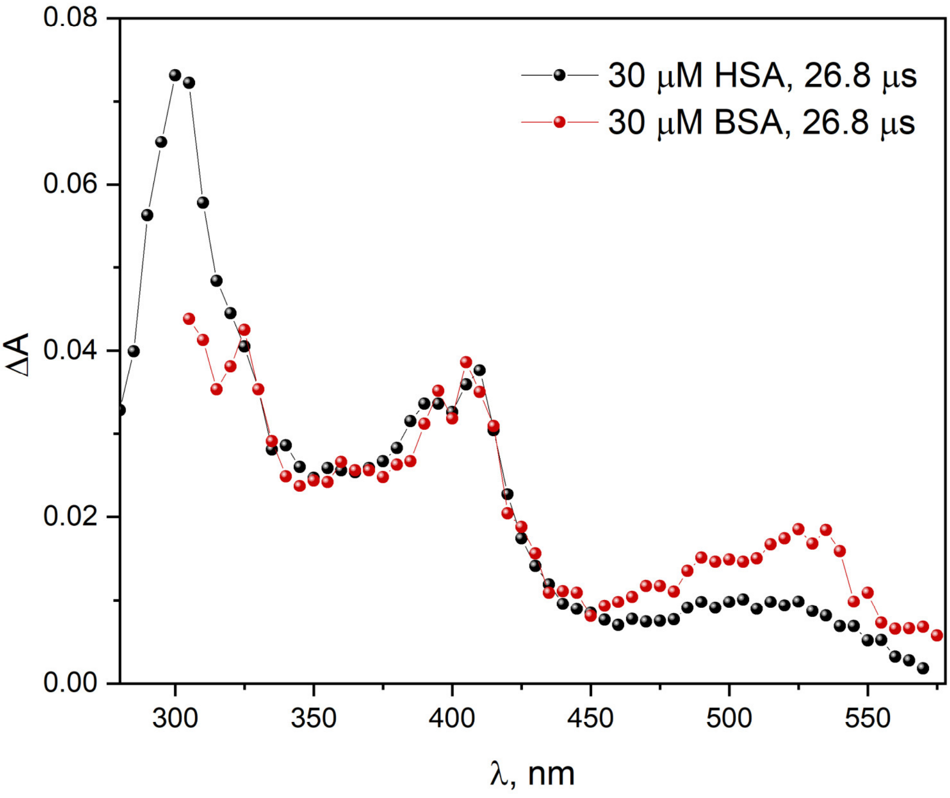

Figure S6. Transient absorption spectra recorded at 26.8 μs time after 4μs pulse irradiation with dose of 800 Gy of N_2O-saturated buffer solutions containing: 30 μM HSA or 30 μM BSA and 0.1M NaN_3. Spectra were normalized.

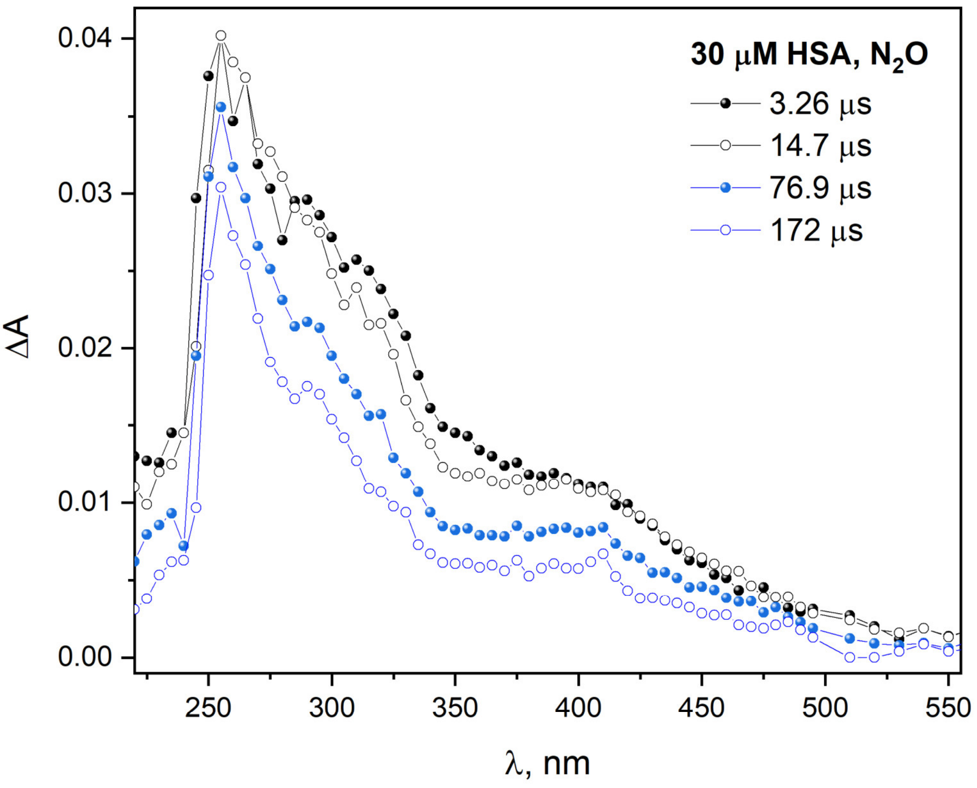

Figure S7. Transient absorption spectra recorded at various time after 17 ns pulse irradiation with dose of 55 Gy of N_2O-saturated buffer solutions containing 30 μM HSA.

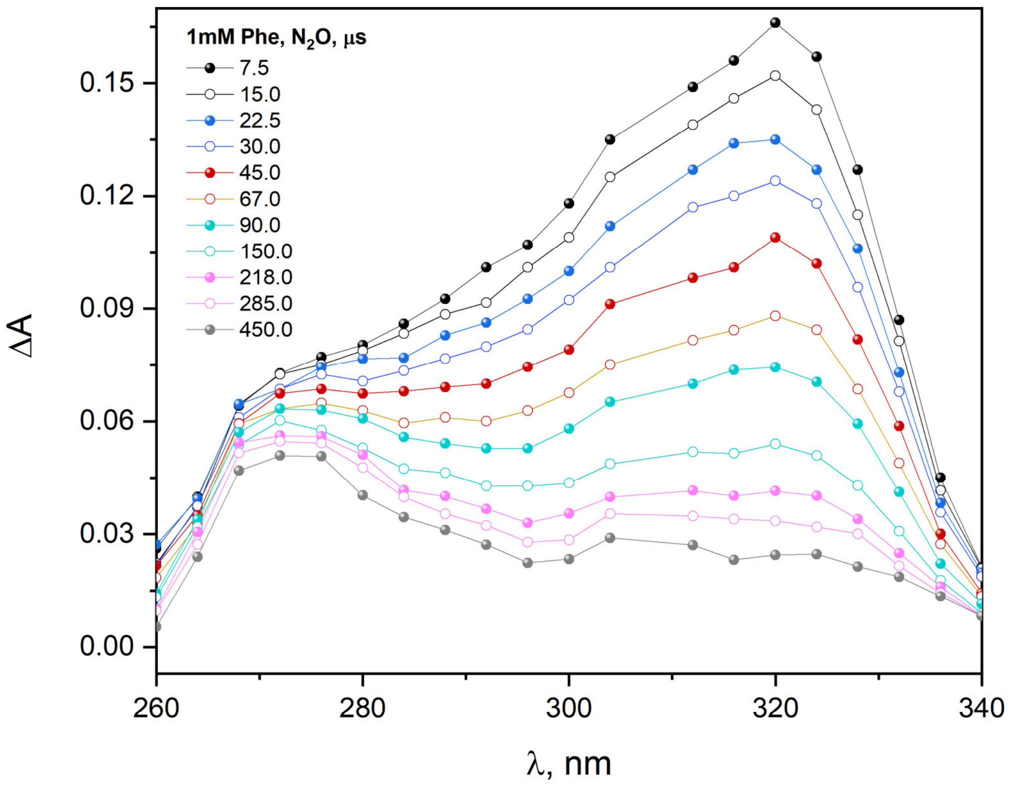

Figure S8. Transient absorption spectra recorded at various time after 17 ns pulse irradiation with dose of 55 Gy of N_2O-saturated buffer solutions containing 1 mM Phe.

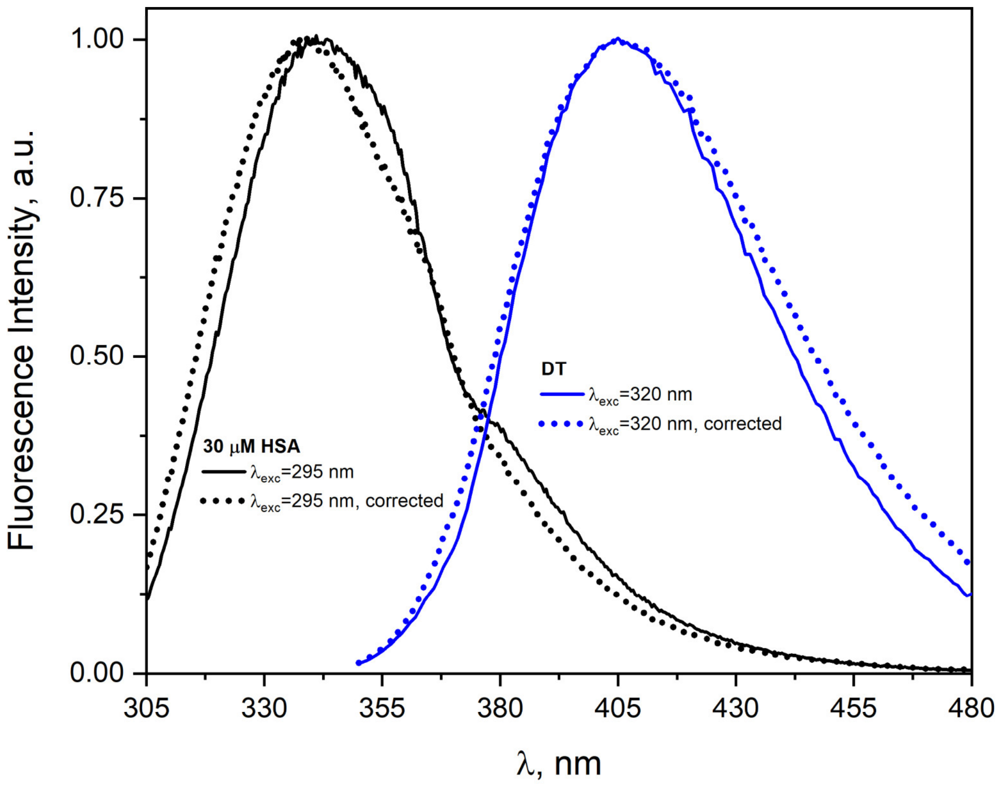

Figure S9. Fluorescence spectra of aqueous solutions of HSA (30 μM), λ_{exc}=295 nm and DT, λ_{exc}=320 nm (generated by irradiation, see caption for Figure 12).
